# “Is this professionally correct?”: understanding the criteria nurses use to evaluate information

**DOI:** 10.5195/jmla.2025.2163

**Published:** 2025-10-23

**Authors:** Brandon Patterson, Anne R. Diekema, Elizabeth (Betsy) S. Hopkins, Duane Wilson, Nena Schvaneveldt

**Affiliations:** 1 b.patterson@utah.edu, Technology Engagement Librarian, Spencer S. Eccles Health Sciences Library, University of Utah, Salt Lake City, UT; 2 annediekema@suu.edu, Assistant Professor, Department Chair, and Instruction and Outreach Librarian, Gerald R. Sherratt Library, Southern Utah University, Cedar City, UT; 3 betsy_hopkins@byu.edu, Nursing Librarian, Harold B. Lee Library, Brigham Young University, Provo, UT; 4 duane_wilson@byu.edu, Assessment Facilitator, Harold. B Lee Library, Brigham Young University, Provo, UT; 5 nena.schvaneveldt@utah.edu, Education Librarian, Spencer S. Eccles Health Sciences Library, University of Utah, Salt Lake City, UT

**Keywords:** Information Literacy, Nurses, Evidence-Based Nursing, Evidence-Based Practice, Evaluation Criteria, Critical Appraisal

## Abstract

**Objective:**

Nurses must evaluate and sift through large quantities of information of varying quality as part of patient care. This study sought to determine nurses' evaluation criteria when encountering health information, including consumer health information written for the general public and scholarly sources, such as journal articles.

**Methods:**

We employed a mixed-methods approach with a survey and follow-up individual interviews. In both the survey and interviews, nurses were asked to evaluate information written for the general public or a scholarly audience. Interviewees were encouraged to think aloud to elucidate their criteria. We analyzed data using descriptive statistics and inductive thematic analysis.

**Results:**

Criteria used for both consumer and scholarly information were similar, with accuracy, relevance, authority, purpose, and currency as the most frequently reported. Nurses often relied on easily identifiable characteristics, such as where information came from, funding sources, intended audience, or its concordance with their prior knowledge. Nurses demonstrated awareness of the need to evaluate methodology in studies, especially empirical studies, for accuracy and relevance. However, they were less likely to evaluate methodology in review articles.

**Conclusions:**

Nurses value accurate, relevant information; however, their evaluation criteria are often superficial. Educators should encourage nursing students to engage more deeply with the nuances of evaluation. While many nurses pointed to research and peer review as evidence of accuracy, fewer demonstrated a deeper understanding of how to evaluate particular research methodologies, such as systematic reviews.

## INTRODUCTION

Knowing how to evaluate or assess information to ensure its quality is an essential skill for nurses at all levels. Nurses are expected to use the best available research evidence combined with their clinical expertise to provide the best care for their patients [1]. This is epitomized by a professional framework known as evidence-based practice (EBP), which is considered a core concept by the American Association of Colleges of Nursing (AACN) for both entry-level and advanced practice nursing education. While entry-level nurses are expected to “evaluate appropriateness and strength of the evidence,” advanced-level nurses also “lead the transition of evidence into practice” (p. 38) [2]. Evaluation is a key component of EBP, and three of the 24 EBP competencies concern evaluation: evaluation of pre-appraised evidence, evaluation of the strength and applicability of research studies, and evaluating and synthesizing a body of evidence [3]. The literature describes the ability to assess and apply information to guide EBP several different ways: information evaluation [4], critical health literacy [5], research appraisal [6], critical appraisal [3], source evaluation [7], information appraisal [8, 9], information credibility sourcing [10], information validation [11], or health information appraisal [5, 12]. Given there is no consistent understanding of this skill, and to aid the interprofessional dialogue, we refer to this collective suite of EBP skills as evaluation of information, or evaluation hereafter.

Evaluation tools and checklists are commonly used in evidence-based practice to capture the complexity of the appraisal of medical information. Some of the criteria evaluated within critical appraisal checklists include the level of evidence, sample size, and random assignment [13, 14]. These tools provide nurses with a consistent approach to use when evaluating evidence. However, critical evaluation checklists often lack standardization across tools and are characterized by a general unwieldiness due to the number of evaluative questions incorporated into a single document [15–17]. Many nurses would benefit from simplified evaluation checklists, as well as training on how to use these checklists effectively [13, 18].

Research shows that many nurses are aware of their struggle with evaluating information and express a need for more instruction and practice [7, 19, 20]. Nursing educators, including health science librarians, are likewise aware of these challenges, with the literature describing how to best teach EBP and different methods for educating nurses on how to evaluate their sources [21–27]. CRAAP [28] and SIFT [29] are two well-known evaluation frameworks. CRAAP encourages novice researchers to consider the following criteria when evaluating a source: currency, relevance, authority, accuracy, and purpose. A known limitation of CRAAP is that evaluations from this framework tend towards binary conclusions, where sources are labeled as either good or bad. By comparison, SIFT (Stop, Investigate, Find Better Coverage, and Trace Claims) is a process-based approach, which encourages researchers to compare and contrast multiple sources against one another and follow claims back to their original sources [29]. While these approaches are popular frameworks for teaching information literacy within secondary and baccalaureate settings, they may be insufficient to evaluate medical information in clinical evidence-based settings. For medical information, criteria are typically expanded with indicators specific to research studies, such as the research methodology, including the level of evidence, sample size, and random assignment, among others [13, 14].

Given the limited time most nursing students spend during their clinicals on reading and discussing research, it is not surprising that in-depth evaluations are out of reach for most nurses unless trained in graduate school [21, 30, 31]. A research-to-practice gap exists where the training nurses receive does not translate easily to the job, impacts the evaluation of research articles, and where nurses fail to see the relevance of research to their professional practice [19, 32]. Nurses gravitate to sources of evidence they previously deemed reliable, and have difficulty evaluating the suitability of research found in new and unknown sources [33]. In their evaluation, nurses often utilize only basic or surface-level evaluation criteria, such as publication date and publisher, rather than content-level criteria such as aspects of the research methodology [7, 8].

The purpose of this work is to investigate what criteria nurses apply in evaluation tasks to uncover insights into the realities of the nursing profession that may be used to inform nursing education. This study explores what criteria practicing nurses utilize when assessing the quality of information and whether information is suitable for informing their clinical practice. This study's design emerged from the following research question: “What are the evaluation criteria used by nurses for consumer and scholarly health information?”

## METHODS

To determine what evaluation criteria nurses use, we utilized a mixed-methods research design, combining data from a survey and 18 individual interviews. The methodological approach is based on Seo [34], who combined survey research with interviews to study how respondents perceived information credibility. The interviews follow the survey to gain a more in-depth understanding of the evaluation criteria used. The research was approved by the Institutional Review Boards of the University of Utah (IRB_00145787), Southern Utah University (IRB #09-112021d), and Brigham Young University (IRB2021-349).

The survey (see Appendix A) started with demographic questions, followed by a scenario to evaluate an abbreviated popular source and scholarly source: “Assume you have the following information to make a clinical decision. Please evaluate it.” The first source was an excerpt from a website, the second was an abstract of an article, and both excerpts had links to the full text should respondents wish to explore more of the source. Both sources discussed flu vaccination and required nuanced judgments to evaluate. After respondents provided their evaluation of the sources, the survey presented a checklist of potential evaluation criteria. Respondents indicated which criteria they typically use to evaluate information as a nurse. Criteria originated from Seo et al. [34], the CRAAP test [28], SIFT [29], and the ACRL standards for nursing [35]. Given the types of materials the nurses would be evaluating, we included a wide variety of criteria. A list of the evaluation criteria used in the survey and their sources can be found in Appendix B.

We disseminated the survey through the Utah Nursing Association and Utah Nurse Practitioners, to nursing program alumni from around the state (including one private university and three public universities), and one health system associated with an academic medical center. The survey was open from January - April 2022. Respondents had at least an LPN license and were currently working as nurses to be eligible. To incentivize participation, we held a drawing for interested respondents to receive one of twenty $20 gift cards.

For our statistical analysis, we used SPSS to run chi-square tests of independence. Because a separate test had to be run against each possible evaluation response, to reduce the chances of a false positive result, we used the Benjamin-Hochberg critical value to adjust the p-value.

At the end of the survey, respondents could express interest in participating in a follow-up interview; they submitted their email addresses and chose the specialties that most closely aligned with their work. Respondents willing to be interviewed were clustered into similar specialties, and a purposive sample from each specialty cluster was randomly selected. Interviewees received a $25 gift card upon completion of the interview. Funding for all study incentives was provided by two of the researchers' universities.

We conducted 18 semi-structured interviews via Zoom in July and August of 2022. Two investigators were present at each interview, one conducting the interview and the other taking notes. Interviewees were randomly assigned to evaluate a popular source or a journal article, both on the topic of nurse burnout, and both published in 2021. As in the survey, the sources selected required a nuanced judgment. Interviewees were asked to think aloud and describe their process when evaluating the information during the interview. A copy of the interview protocol may be found in Appendix C. Further explanation of the sources used in the scenarios for the survey and interviews may be found in Appendix D.

We used Zoom's auto-transcribe feature to generate initial interview transcripts, then cleaned and checked the transcripts for accuracy. We then followed an inductive coding strategy to identify themes in the interview data to develop a codebook using a sample of two interviews - one where the interviewee evaluated the website and another where the interviewee evaluated a research article. All four researchers examined the sample transcripts for emerging themes, and we came to a consensus codebook consisting of 29 lead codes and 60 granular codes (Appendix E). The remaining transcripts were separately coded by two individual researchers using the codebook. Researchers reconciled any differences in their coding by discussion. A third researcher was brought in to reconcile when a consensus between the two researchers could not be reached. Data associated with this article are available; see the data availability statement at the end of this article.

## RESULTS

The survey had 344 eligible respondents, with many specialties, levels of education, and years of experience represented (Table 1). We asked respondents about their specialties to ensure we collected data from a diverse group of nurses and workplaces. Respondents could select more than one option, so the number of selections exceeds the total number of responses. Each of the 18 listed specialties was selected at least once, with “other” specialties including nursing educator, pain management, and geriatric nurse (see Appendix F). Upon coding and reconciliation, the interviews yielded 423 instances of codes related to evaluation criteria.

**Table 1:** Respondent demographic data: highest degree of educational attainment, years' experience as a licensed nurse, and areas of clinical specialty

**Respondents' highest level of educational attainment (N=344)**		
**Education Level**	**Frequency**	**Percent of Respondents**
LPN	2	0.6%
RN	42	12.2%
BSN	209	60.8%
MSN	66	19.2%
DNP	19	5.5%
PhD	6	1.7%
**Respondents' years working as a licensed nurse (N=344)**		
**Years Worked**	**Frequency**	**Percent of Respondents**
0-5 years	108	31.4%
6-10 years	57	16.6%
11-15 years	38	11.0%
16-20 years	35	10.2%
21-25 years	27	7.8%
26-30 years	21	6.1%
31-35 years	15	4.4%
36-40 years	22	6.4%
41+ years	21	6.1%
**Respondents' clinical specialty within nursing. Respondents could select more than one option, so number of selections exceeds total number of responses (N=344).**		
**Clinical Specialty**	**Frequency**	**Percent of Respondents**
Medical-surgical	58	16.9%
Other	58	16.9%
Administration (e.g. charge nurse, case management, nurse manager)	50	14.5%
Critical care	49	14.2%
Pediatrics	36	10.5%
Ambulatory care	31	9.0%
Emergency	31	9.0%
Neonatal care	26	7.6%
Psychiatric/mental health	26	7.6%
Labor & delivery/midwifery	25	7.3%
Maternity	22	6.4%
Perioperative	20	5.8%
Cardiac care	18	5.2%
Palliative care/hospice	17	4.9%
Oncology/hematology	15	4.4%
Public health	14	4.1%
School (K-12)	14	4.1%
Disease-specific	8	2.3%
Wound/ostomy/continence	6	1.7%

### Overview of common evaluation criteria

The most common evaluation criteria used in the survey and those observed in the interview data can be found in Table 2. The criteria labels had to be consolidated because the survey provided a list of criteria for respondents to choose from, while the interview data was coded inductively, allowing criteria to emerge from the data.

**Table 2:** Most common evaluation criteria from the survey choices and interview codes.

Consolidated label	Survey choice(s)	Interview code(s)	Description
Accuracy (including prior knowledge)	Accuracy, Fits Prior Knowledge	Accuracy, Accuracy - Fits Prior Knowledge	The correctness of the information (citations present to support claims, possible bias, editorial process/peer review, matches existing research or prior knowledge).
Methodology	N/A	Methodology	Details on how the study or research was completed.
Currency	Currency	Currency	The age of the information in relation to the information need or research topic.
Authority	Author's expertise	Authority	The qualifications/education or reputation of the author(s), journal, publisher, or organization.
Relevance	Relevancy	Relevance	Fit between the research question/information need, and the content of the source.
Purpose	Purpose, Bias, Financial Backing	Purpose, Purpose - Funding; Purpose - Persuade	References to why the information was created and for which audience. Includes bias and funding.
Publisher, production, or dissemination	Production and/or dissemination	Publisher	References to the publisher, how it was produced, or disseminated.

**Figure 1:**
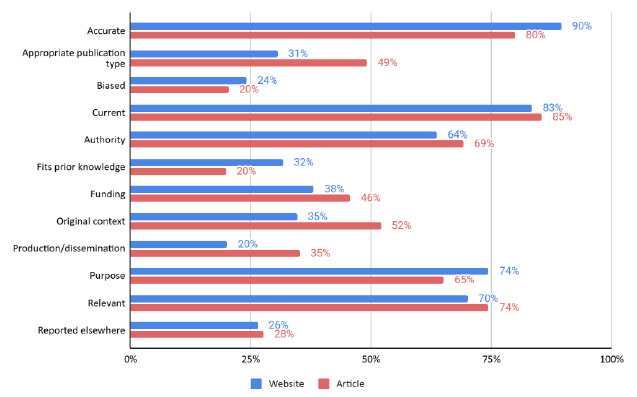
Percentage of respondents from the survey reporting evaluation criteria for the website and article (N=344)

In the survey checklists, nurses selected similar criteria for both the website and journal article, with currency and accuracy selected most frequently. Less important was whether the information fit the nurse's prior knowledge (especially for journal articles), bias, and the information's production or dissemination on websites. Figure 1 shows the evaluation criteria prevalence for websites and articles.

Notably, only a few survey respondents clicked the link to see the complete information source and its context. Nearly a quarter of respondents (n=80, 23%) clicked a link to see the full text of either the website (n=79, 23%) or the journal article (n=51, 15%). Most respondents who looked at the full text of one source looked at both (n=47/80, 59%). Similar criteria prevalence were reported in the website and article, as seen in figure 1.

The interviews followed a similar pattern. Of all the criteria codes (n=423) shown in table 3, accuracy was the most frequent code (n=88, 21%). Authority and methodology were the next most frequent, each with 13% of total codes counted (n=66), followed by purpose and relevance, each with 55 (13%). In total, four of the top five codes, representing nearly two-thirds of all criteria codes (n=264, 62%), represent all but one criteria in the CRAAP checklist: relevance, accuracy, authority, and purpose.

**Table 3:** Criteria codes found in interviews

Code count of criteria found in interviews (n = 423)		
Code Name	Frequency	Percent found
Accuracy	88	20.7%
Authority	66	15.5%
Methodology	66	15.5%
Purpose	55	12.9%
Relevance	55	12.9%
Currency	33	7.8%
Miscellaneous	26	6.1%
Editorial Quality	17	4.0%
Publisher	11	2.6%
Undetermined	8	1.9%

After looking at the evaluation criteria in the survey responses and in the coded interview transcripts separately, we looked for overlap between the two. To compare rates in a meaningful way, we report the data as percentages rather than counts because the interview population (n=18) is much smaller than the survey population (N=344). Survey respondents looked at both a website and a research article, but interviewees only evaluated one or the other, so analysis for interviews is split into interviewees who evaluated the website (n=9) and those who evaluated the research article (n=9). First, we aligned the survey checklist criteria with the interview codes. Then, we determined whether the criteria in the survey checklist were present at any point during the interviews. Granular codes used to code the interviews were conflated into their top-level codes to facilitate comparison with the survey evaluation criteria. Further detail is delineated in table 2.

**Figure 2:**
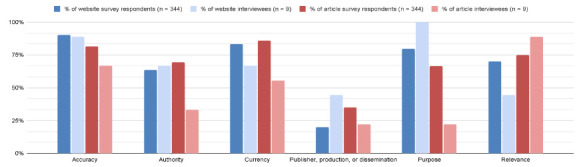
Website and article evaluation criteria present in surveys and interview responses

Website evaluation criteria were present at differing rates in the survey and the interviews. Interviewees were more likely to discuss dissemination or publication in interviews than in the survey, and they were far more likely to discuss purpose as a criterion for website evaluation. Interviewees were less likely to discuss currency or relevance than survey respondents. Comparisons between website evaluation criteria of survey respondents or interviewees are found in figure 2.

Journal article evaluation criteria showed more discrepancies between survey responses and interviews. Far fewer interviewees than survey respondents discussed an article's purpose, authority, or currency, and fewer interviewees discussed publisher, production, or dissemination and accuracy. More interviewees discussed relevance than the survey results. Comparisons between journal article evaluation criteria of survey respondents or interviewees are found in figure 2.

To determine if there was a statistically significant difference between nurses' education level, number of years worked, and the criteria the nurses chose for evaluation, we ran chi-square tests of independence for each of these demographics and each criterion that the nurses might have chosen. After adjusting the p-value for false positives, we found that none of these factors were statistically significant (see Appendix G for statistical analysis).

We also wanted to know if entry-level nurses (LPN, RN, BSN) answered differently than advanced practice nurses, nursing faculty, or nurses with otherwise advanced education (MSN, DNP, PhD). We recoded the variables and ran chi-square tests of independence on this vs how the ratings were completed. After adjusting for false positives, there were no statistically significant differences. Finally, we sought to determine if nurses who selected administration as a specialty answered differently. After adjusting the p-value for false positives, we found that there were no significant differences (see Appendix G for statistical analysis).

While the survey provided the preceding insights into nurses and the evaluation criteria they use, the interviews provided richer data, which is highlighted in the following discussion of how nurses conceptualized specific evaluation criteria.

#### Accuracy

Interviewees often established accuracy by checking for the presence of citations and whether the source was peer reviewed. Nurses also used their existing knowledge and clinical experience to judge the reliability of new information. One participant asked, “Did it match up again with my nursing knowledge?” and another referred to evaluating information “on instinct and my own training.”

#### Methodology

Respondents typically mentioned this criterion when referring to a scholarly source. Interviewees mentioned some specific aspects of methodology, including sample size, population, and study design, as in this statement: “Is that based on a good study that was…randomized and controlled?” Interviewees also discussed confounding factors and levels of evidence. For example, one stated: “evaluating the levels of evidence, and where those lie in determining how strong the evidence can be.” In other cases, nurses mentioned the type of study as a criterion, such as “a lot of them were just case studies.”

#### Currency

In interviews, currency was mentioned roughly half as often as relevance, authority, and purpose. The articles shown to all respondents and interviewees were all within two years old, and the topics were such that currency is embedded in the subject's relevance. When bringing up criteria related to currency, nurses often noted the year the article was written, with several writing off information if it was over five years old: “If it was older than five years? I probably wouldn't give it much thought.” Some spoke to the field's rapid evolution as a reason to value currency.

#### Authority

In most cases, nurses discussed the authority of the author: “Who is this doctor that's writing this article? What are his credentials?” Several respondents mentioned the reputation of the journal, website, or organization, specifically mentioning the publisher of the content, i.e., the Mayo Clinic. Nurses often equated authority with trust, and, generally, there seemed to be a distrust of news or news agencies, with one interviewee stating, “I trusted it more than if I would have just read it in a newspaper.” Instead, nurses favored reputable national organizations, credentials, or educational backgrounds attached to the author. The option given to respondents of the survey was “author's expertise,” which alluded to authority, and no respondent decided to expand upon this in the open-response questions.

#### Relevance

Relevance often included discussions of the information source's population, the nurse's role, and how well the information matched the nurse's circumstances, including their specialization or patient population. One interviewee summarized it well: “Nursing is just such a practical field that if it doesn't help me right here, right now, or it doesn't help me move in a positive direction, then it's not worth my time.” As an artifact of the international contexts of the research articles nurses were asked to evaluate in the study, many nurses commented that the article might not be relevant given the differences in healthcare systems between countries.

#### Purpose

When discussing purpose, interviewees often mentioned bias, funding source, and intended audience. One executive discussed medical device or pharmaceutical companies funding research: “A big one, when you talk about surgical [research] is, did a pharmaceutical or a medical device [company] fund it? I start to really question the research if that's the case.” Advertisements were seen as a marker for a lower quality or non-scholarly source, and were present in the website that interviewees evaluated. The intended audience was often judged by the way a source was written. While sensationalizing and other methods of getting the reader's attention were often noted, one nurse joked that “if it's boring, it's gotta be true.” However, when evaluating information intended for patients, a website's familiar tone was favorably remarked on as, “worded well to help the general public understand the information.”

#### Other criteria

Most miscellaneous criteria coded from the interviews were related to the source type or the container of the information, such as a journal article or blog. Overall, nurses were favorable toward research, with several viewing a research study as a criterion or marker of quality rather than taking a more nuanced view. Finally, external factors influenced the evaluation criteria. A need for concise material due to time constraints or organizational guidelines concerning information for patients. Several nurses wanted the information presented to them to contain actionable solutions that they could apply, such as this interviewee who reacted to the website as, “it addresses causes but doesn't really suggest any solutions.”

#### Differences in criteria in the interviews by type of source: consumer vs. scholarly

**Figure 3:**
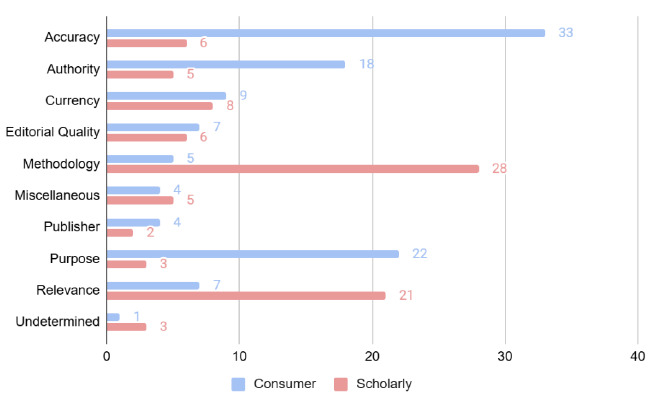
Consumer vs. scholarly criteria comparison (n=199)

Interviewees evaluated either a website (consumer) or a journal article (scholarly). We compared the evaluation criteria for the two source types. To facilitate this, we examined the 199 codes noted during the narration portion of the interviews (figure 3). The criteria mentioned by interviewees naturally varied depending on what type of source they were evaluating. Interviewees evaluating the article mentioned methodology much more. Interviewees mentioned accuracy, authority, and purpose much more when evaluating the consumer source. One counterintuitive finding is that relevance was mentioned much more by those evaluating the scholarly source. The article we chose, a study completed in China, elicited comments from nurses about its relevance to their populations in the Intermountain West.

## DISCUSSION

We set out to determine what criteria working nurses use to evaluate consumer and scholarly health information and how this might inform nursing education. We found that education level, number of years worked in the field, and clinical specialty had no bearing on differentiating the evaluation skills of nurses. When evaluating information, nurses consider accuracy, authority, currency, methodology, purpose, and relevance most frequently. Nurses mostly use surface-level criteria to evaluate information. The majority of their criteria reflect those in the easily remembered and frequently taught CRAAP test [28]. This echoes the findings of Holliday and Rogers that the way information literacy is framed may impact the way learners engage with it [36].

If we present evaluation as a simple task of engaging with criteria, learners are less likely to move beyond this list to a more sophisticated understanding, which includes critically analyzing the methodology and the implications of the paper's findings. Another important element of this process involves considering what changes might be necessary to the nurses' current knowledge, based on the evidence at hand.

One salient example of surface-level criteria is the general tendency among study participants to believe journal articles over websites. The “container” in which information is housed can provide helpful context clues about the source [17]. Peer review is designed to ensure quality and accuracy, so the preference for the journal article is understandable. However, by relying on external factors like source type, nurses can miss underlying problems with the research study itself. Nurses discussed criteria for evaluating research, such as sample size and methodology, but lacked knowledge about systematic review methodology. Even sample size and methodology type are surface-level characteristics; nurses probably use these criteria because they are easy to see in an article, instead of a more time-intensive yet essential process that involves complex reading and analysis of study design. In-depth knowledge about research and publishing processes is necessary for nurses to complete a competent evaluation of these resources.

The ways nurses establish accuracy were also superficial and prone to error. For example, the presence of citations alone is not an indicator that the information is accurate. In his SIFT model, Caulfield urges readers to examine the original context as it can be misrepresented [29]. This issue is confounded when current AI technology often fabricates citations to non-existent research studies [37]. Another area of concern was nurses' tendency to accept information that affirms their current knowledge, a practice that leads to confirmation bias. This finding has significant implications for the provision of healthcare and the adoption of new evidence into clinical practice.

Some participants seemed puzzled by our use of the terminology “evaluation of information”. One explanation is that critical appraisal, a more detailed process for determining the merit of studies, may seem disconnected from evaluation criteria commonly taught in earlier education. This can be further obfuscated by inconsistent terminology. Evaluation, fact-checking, critical appraisal, judgment, quality, and literacy are all terms in the literature about the criteria used to determine information's usefulness and quality.

From these findings, it is clear that nurses need deeper preparation for evaluating information in today's complex knowledge environment. Through our research, we discovered that education level and years of experience didn't impact the criteria used by nurses to evaluate information, indicating a widespread problem in the profession. Health sciences librarians have a responsibility to make a difference here, both for students at their individual schools and perhaps more broadly as a profession.

Health sciences librarians need to help nursing students and nurses move past the easy criteria they are currently applying to the more challenging realm of critical analysis. Critical appraisal and more complex evaluation techniques should be connected explicitly to and build on previously learned evaluation criteria. Providing examples of unexpected authority or credibility, such as when Wikipedia pages are reliable or journal articles face retraction, may help nurses practice operating in authentically ambiguous situations. Effective methods for fact-checking claims, such as lateral reading proposed by Caulfield's SIFT model [29, 38], can provide additional tools. Spending more time discussing health research may help students and nurses familiarize themselves with research and the process of appraisal, leading them to more readily evaluate the information they encounter [19, 21]. Ideally, this would be accomplished in collaboration with nursing faculty, who can better speak to the authenticity of specific scenarios, building realistic cases. From there, librarians can provide guidance on evaluating the information to solve problems nursing students and nurses face, rather than restricting education about appraisal to an assignment or classroom activity. This may facilitate greater transfer and practice of these necessary skills, which Scott and colleagues recommend should be taught, complementing clinical practice, not in isolation [23].

Sophisticated instruction is more time-consuming than a one-off lecture about the CRAAP acronym, and nurses have expressed that they want and need more practice and instruction with these concepts [7, 19, 20]. Nurses and nursing students can improve their research appraisal skills long term with the right intervention [21, 22]. Clinical librarians may wish to work with continuing education to allow practicing nurses to further refine and refresh their evaluation skills. This is especially important as nursing is the largest healthcare profession in the United States, with an exceptionally wide scope of practice [39]. Nurses' ability to accurately evaluate information impacts their practice, as well as their advice and advocacy to patients.

Health sciences librarians may also have a role in working with accreditation bodies on issues related to information evaluation. It is apparent that nurses are falling below the relevant standards. However, previous studies have found that librarians are not always involved in nursing education [40] and that faculty tend to teach to the disciplinary standards [41]. By continuing conversations with nursing educators, especially those involved in developing accreditation standards, librarians can continue advocating for nurses to develop more sophisticated skills in evaluation. Additionally, advocacy from organizations, such as the Medical Library Association, to promote the role of health sciences librarians as collaborators in instruction for EBP may help advance the conversation more broadly.

## LIMITATIONS AND FUTURE WORK

Our study had several limitations. We recognize that we may have influenced nurses' responses in selecting sources for them to evaluate. The survey sources discussed vaccines, a controversial topic lending itself to strong opinions, which may have impacted the reporting of bias. The interview sources were recent, which may have affected how often interviewees remarked on their currency. Additionally, we intentionally chose a systematic review in the survey because it was considered “good evidence” by its design, which could impact respondents' reporting of methodology as a criterion.

The construction of the criteria checklist on the survey may also have some limitations. Due to the heterogeneity of terminology in the literature to capture the complex phenomenon of evaluation, we considered criteria from several sources. The conglomeration of multiple concepts could have led to confusion. Also, we used the subject-specific ACRL standards for nursing, instead of the broader, more up-to-date ACRL Framework. We felt the subject-specific nature of the ACRL standards was more relevant to our study but our criteria were missing the concepts present in the Framework.

Our study was broad in scope, and more research is needed to fully understand how nurses evaluate information. Studies on how nurses apply critical appraisal techniques for scholarly sources and how nurses approach consumer health sources specifically would help fill in the details. A comparison between the evaluation criteria nurses use and those found in educational standards and common learning objectives might also contribute to a better understanding of the terminology disconnect and the research-to-practice gap. Also, it is important to understand how the concepts in the ACRL Framework show up in nurses' information evaluation practices, if at all. Further research may seek to better understand the ways different groups of nurses engage with different evaluation tools; for example, determining if nurses use specific critical appraisal tools for scholarly articles, or comparing nurses' practices with educational standards. Additional research into the evaluation practices of groups of nurses by type, such as entry-level nurses or nurse researchers, could also yield more specific insights for those particular groups.

## Data Availability

Data associated with this article are available in the Hive Data Repository at https://doi.org/10.7278/S5d-908e-j35w.
