# Supplementary material for: “Is this professionally correct?”: understanding the criteria nurses use to evaluate information
Source: J Med Libr Assoc. 2025 Oct 23;113(4):298–309. doi: 10.5195/jmla.2025.2163 (PMC12604069; doi:10.5195/jmla.2025.2163)
Supplement: Supplementary file 1 — Appendix A [file jmla-113-4-298-s01.docx]

**Appendix A: Survey instrument**

**Nurses' Evaluation of Information Sources**

To participate, we need your permission. Here is information about the survey:

The purpose of this research study is to identify evaluation criteria and processes used by nurses for consumer and scholarly health information. The aggregated results from this survey will be shared among the investigators, will be added to an open online data repository, and may appear in presentations and academic journal publications. 

The survey takes approximately 10-15 minutes to complete and includes demographic as well as multiple-choice questions with options to write in additional information. All study participants will remain anonymous. Participation in this survey is completely voluntary. You may end participation in the survey at any time without penalty. There are no foreseeable risks involved in participating in this study other than those encountered in day-to-day life. 

Should you choose to enter your name into a drawing for a $20 Amazon gift card at the end of the survey, you will be taken to a completely separate form to provide your information. There will be no link between your survey responses and your name and contact information. No information that could identify you will be shared in publications about this study. You will not be paid for participating in this study. There is no cost to participate in this study. 

At the end of this survey, you will have an opportunity to volunteer for an optional online interview at a later date. If you are selected to participate in a follow-up interview, you will receive a $25 Amazon gift card.

This study has been approved through the Institutional Review Boards of the University of Utah (IRB_00145787), Southern Utah University (IRB #09-112021d), and Brigham Young University (IRB2021-349). If you have questions or concerns: The Principal Investigator

 Survey requirements are that you are at least 18 years of age, that you are currently working as a nurse, and that you have at least an LPN license. By selecting “I agree,” you are saying you:
-Have read the above information
-Voluntarily agree to participate
-Are 18 years of age or older 

| 1. | **About this survey** |
| --- | --- |
| 2. | By selecting “I agree,” you are saying you have read the above information, voluntarily agree to participate, are 18 years of age or older.   - I agree - I do not agree |
| 3. | **Demographics** |
| 4. | What is your highest level of nursing education?   - CNA - LPN - RN - BSN - MSN - DNP - PhD |
| 5. | How many years have you worked as a nurse?   - 0-5 years (1) - 6-10 years (2) - 11-15 years (3) - 16-20 years (4) - 21-25 years (5) - 26-30 years (6) - 31-35 years (7) - 36-40 years (8) - 41+ years (9) |
| 6. | What option(s) most closely aligns with your clinical specialty?   - Administration (e.g. charge nurse, case management, nurse manager) (1) - Ambulatory care (2) - Cardiac care (3) - Critical care (4) - Disease-specific (5) - Emergency (6) - Labor & delivery/midwifery (7) - Maternity (8) - Medical-surgical (9) - Neonatal care (10) - Oncology/hematology (11) - Palliative care/hospice (12) - Pediatrics (13) - Perioperative (14) - Psychiatric/mental health (15) - Public health (16) - School (K-12) (17) - Wound/ostomy/continence (18) - Other (19) ________________________________________________ |
| **Scenario: Website evaluation** | |
| 5. | Assume you have the following information to make a clinical decision. Please evaluate it. Swanson, W. S. (2020). Colds and flu: prevention and treatment tips. Knowyourotcs.org. <https://www.knowyourotcs.org/tips-for-cold-and-flu/>  "I always like to start by saying, the flu shot is your best shot at preventing an influenza infection. While it’s best to get immunized early in the flu season, we often continue to recommend getting the flu vaccine well into May. Call you doctor’s office, go to your local pharmacy, or use the CDC’s Vaccine Finder to locate the vaccine available in your zip code. Remember, if you have a baby, they will need two doses (separated by 28 days or more), and if your child is under the age of nine and they have never had the flu vaccine before, they’ll need two doses this year, too!"   - This is bad information - This is not very good information - This information is neutral or mixed - This is pretty good information - This is good information - I am not sure |
| 6. | Please explain why. (open ended) |
| **Scenario: Article evaluation** | |
| 7. | Assume you have the following information to make a clinical decision. Please evaluate it.  Cagle, P. J. (2021). Shoulder injury after vaccination: A systematic review. Revista Brasileira de Ortopedia, 56(3), 299-306. https://doi.org/10.1055/s-0040-1719086  "Adverse reactions to vaccine injections are usually mild and incredibly rare in nature, but multiple cases of shoulder events including bursitis, generalized pain or decreased range of motion have been reported following routine vaccine administrations. These events are known as Shoulder Injury Related to Vaccine Administration or SIRVA.  A systematic review of literature was performed to identify all published accounts of SIRVA. Twenty-seven papers reporting one or more accounts of SIRVA were identified. The most common vaccination involved was the Influenza vaccine. The most common symptoms were pain that began in 48 hours or less and loss of shoulder range of motion. The most common treatment modalities were physical therapy, corticosteroid injections and anti-inflammatory medication; but in some patients, surgery was required. Regardless of intervention, the vast majority of outcomes demonstrated improved pain and functional except in the occasions of nerve injury.  The etiology of SIRVA injuries has multiple possibilities including needle length, mechanical injury from needle overpenetration and the possibility of an immune inflammatory response from the vaccine components, but a unique definitive test or quantifiably result does not yet exist."   - This is bad information - This is not very good information - This information is neutral or mixed - This is pretty good information - This is good information - I am not sure |
| 8. | Please explain why. (open ended) |
| **Criteria checklists** | |
| 9. | When you evaluate information from **health websites focused on the general public**, what do you pay attention to in your role as a nurse? Check all that apply.   - The information is accurate - The information is relevant to what I need - The information is reported somewhere else - The information is in its original context - The information’s purpose (e.g. presenting facts or opinions) - The information is in the most appropriate publication type - The information’s production and/or dissemination - The information fits with what I already know - The information is biased toward one point of view - The information is current - The source’s financial backing, financing, or underwriting - The authors’ expertise - None of the above |
| 10. | When you evaluate health information from scholarly sources, which of these factors do you pay attention to in your role as a nurse? Check all that apply.   - The information is accurate - The information is relevant to what I need - The information is reported somewhere else - The information is in its original context - The information’s purpose (e.g. presenting facts or opinions) - The information is in the most appropriate publication type - The information’s production and/or dissemination - The information fits with what I already know - The information is biased toward one point of view - The information is current - The source’s financial backing, financing, or underwriting - The authors’ expertise - None of the above |
| **Further participation** | |
| 11. | Please indicate your interest in entering the drawing or interview participation   - I would like to enter the gift card drawing only. - I would like to enter the drawing and volunteer for an interview. - I would like to volunteer for an interview only. - Please take me to the end of the survey. |
