# Supplementary material for: “Is this professionally correct?”: understanding the criteria nurses use to evaluate information
Source: J Med Libr Assoc. 2025 Oct 23;113(4):298–309. doi: 10.5195/jmla.2025.2163 (PMC12604069; doi:10.5195/jmla.2025.2163)
Supplement: Supplementary file 2 — Appendix B [file jmla-113-4-298-s02.docx]

## **Appendix B: Checklist criteria in survey and their sources**

| **Criterion** | **ACRL Nursing** | **CRAAP** | **Seo et al.** | **SIFT** |
| --- | --- | --- | --- | --- |
| Information is accurate | Y^^[[1]](#footnote-0)^^ | Y (accuracy) | Y (“How accurate do you think the story is?”) | Y (Find better coverage) |
| The information is relevant to what I need | Y^^[[2]](#footnote-1)^^ | Y (relevancy) | N | N |
| The information is reported somewhere else | Y¹ | N | N | Y (Find better coverage) |
| The information is in its original context | Y^^[[3]](#footnote-2)^^ | N | N | Y (Trace claims to context) |
| The information’s purpose (e.g. presenting facts or opinions) | Y¹ | Y (purpose) | Y (How would you describe the information in the story (helpful, persuasive, believable)) | Y (Investigate the source) |
| The information is in the most appropriate publication type | Y^^[[4]](#footnote-3)^^ | N | N | N |
| The information’s production and/or dissemination | Y^^[[5]](#footnote-4)^^ | N | N | N |
| The information fits with what I already know | Y^^[[6]](#footnote-5)^^ | N | N | N |
| The information is biased toward one point of view | Y¹ | Y (purpose) | Y (How would you describe the author (can be trusted, biased, fair, tells whole story, factual)) | N |
| The information is current | Y¹ | Y (currency) | N | N |
| The source’s financial backing, financing, or underwriting | N | Y (purpose) | N | N |
| The authors’ expertise | Y¹ | Y (author) | Y (How would you describe the author (can be trusted, biased, fair, tells whole story, factual)) | Y (Investigate the source) |

ACRL Nursing Standards Summary Used:

**Notes 1-3 use Standard Three:**

The information literate nurse critically evaluates the procured information and its sources, and as a result, decides whether or not to modify the initial query and/or seek additional sources and whether to develop a new research process.

**Relevant performance indicator:**

Selects information by articulating and applying criteria for evaluating both the information and its sources.

**Relevant outcome(s):**

- Examines and compares information and evidence from various sources in order to evaluate reliability, validity, accuracy, authority, currency, and point of view or bias.
- Recognizes the cultural, historical, physical, political, social, or other context within which the information was created, and understands the impact of context on interpreting the information.

**Relevant performance indicator:**

Compares new knowledge with prior knowledge to determine the value added, contradictions, or other unique characteristics of the information.

**Relevant outcome:**

- Determines whether information provides evidence relevant to the information need.

**Notes 4-6 use Standard One:**

The information literate nurse determines the nature and extent of the information needed.

**Relevant performance indicator:**

Identifies a variety of types and formats of potential sources for information.

**Relevant outcomes:**

- Identifies likely type of publication where appropriate information is published (e.g., popular vs. trade vs. scholarly, current vs. seminal, primary vs. secondary vs. tertiary).
- Identifies the value and differences of potential resources in a variety of formats (e.g., multimedia, database, website, data set, audio/visual, book, graph).

**Relevant performance indicator:**

Has a working knowledge of the literature in nursing related fields and how it is produced.

**Relevant outcome:**

- Recognizes how scientific, medical, and nursing practice information is formally and informally produced, organized, and disseminated.

1. III.2.5 Standard Three, Performance Indicator Two, Outcome Five: “Examines and compares information and evidence from various sources in order to evaluate reliability, validity, accuracy, authority, currency, and point of view or bias.” [↑](#footnote-ref-0)
2. III.4.6 Standard Three, Performance Indicator Four, Outcome Six: “Determines probable accuracy by questioning the source of the information, limitations of the information gathering tools or strategies, and the reasonableness of the conclusions.” [↑](#footnote-ref-1)
3. III.2.6 Standard Three, Performance Indicator Two, Outcome Six: “Recognizes the cultural, historical, physical, political, social, or other context within which the information was created, and understands the impact of context on interpreting the information.” [↑](#footnote-ref-2)
4. I.2.3 Standard One, Performance Indicator Two, Outcome Three: “Identifies likely type of publication where appropriate information is published (e.g., popular vs. trade vs. scholarly, current vs. seminal, primary vs. secondary vs. tertiary) and Outcome Five: “Identifies the value and differences of potential resources in a variety of formats (e.g., multimedia, database, website, data set, audio/visual, book, graph).” [↑](#footnote-ref-3)
5. I.3.1 Standard One, Performance Indicator Three, Outcome One: “Recognizes how scientific, medical, and nursing practice information is formally and informally produced, organized, and disseminated.” [↑](#footnote-ref-4)
6. Standard Three, Performance Indicator Four: “Compares new knowledge with prior knowledge to determine the value added, contradictions, or other unique characteristics of the information.” [↑](#footnote-ref-5)
