# Supplementary material for: “Is this professionally correct?”: understanding the criteria nurses use to evaluate information
Source: J Med Libr Assoc. 2025 Oct 23;113(4):298–309. doi: 10.5195/jmla.2025.2163 (PMC12604069; doi:10.5195/jmla.2025.2163)
Supplement: Supplementary file 3 — Appendix C [file jmla-113-4-298-s03.docx]

**Appendix C: Interview protocol**

Warmup:

Before we start, please tell me a bit about your work.

Thanks for participating in this interview. My name is [name], and I am the [job title, institution]. [Other researcher] And I am [name, title, institution]. You are being invited to participate in this research study about nurses’ evaluation of information sources. My research team and I are interested to learn more about the criteria and processes nurses use to evaluate information. Being in this study is optional.

If you choose to be in the study, you will be asked to complete an interview that should take approximately 45 minutes of your time. This interview is being conducted via Zoom and you are welcome to leave your video on or turn it off.

You can skip questions that you do not want to answer or stop the interview at any time. The interview recordings will be destroyed after transcription and the transcripts will be de-identified. Interview data will be shared among the investigators, and added to an open online data repository, and may appear in presentations and academic journal publications.

By continuing forward with the interview process, you indicate that you have read the consent documentation and that you agree to participate in this research study.

Any questions before we begin?

INTERVIEW QUESTIONS

30 - 45 minute interviews

INTRODUCTION (5 minutes)

1. Introduction of interviewer & the study
   1. The research question we’re trying to answer is: What are the evaluation criteria and evaluation processes used by nurses for consumer and scholarly health information?
   2. Let’s define some of those terms.
   3. For this study, we are defining consumer health information as “information on health, medical conditions, and medical topics available to the general public, at the lay person level.” (Mt. Sinai LibGuide)
   4. We are defining scholarly health information as “information written primarily for health professionals, scientists, or researchers in the medical and nursing fields, which may not be available to the public and is written at a more specialized, professional level.”
   5. In this study, we are defining “information” as evidence or knowledge that may be found in a variety of places. When we ask you to evaluate the information, we are primarily asking you to look at it regardless of where you would find it. For example, you can find similar information in a website or a journal article, and it can be true or false or misleading in any format. We know that characteristics of the source may inform your judgment of that information, and you can tell us those as well, although we’re mostly concerned with the quality of the information.
2. So, reflecting on those definitions, I’m going to give you a question and feel free to just talk through it. Tell me about the last time you used information in your work and how you evaluated it?
   1. Follow up - what were you using it for?

SCENARIO (10-15 minutes)

1. I am going to give you a scenario for which you need to evaluate some information. Please think aloud, discussing your process for evaluating it. For example please include details like “I am now reading the title” and describe your reaction and thoughts.

Ever since the pandemic you have been feeling a bit worn out on the job and decide to research about burnout. How would you evaluate the following information?

[interviewer shares screen to show interviewee either “[The Biggest Causes of Nurse Burnout and What You Can Do](https://www.mywellbeingindex.org/blog/the-biggest-causes-of-nurse-burnout-and-what-you-can-do)” (consumer source) or “[Impact of burnout, secondary traumatic stress and compassion satisfaction on hand hygiene of healthcare workers during the COVID-19 pandemic](https://doi.org/10.1002/nop2.786)”. Half of interviewees will receive the popular source, and half the scholarly source. The researchers will randomly assign each interviewee to a source before the interview.]

- 1. Tell me about your assessment of this information.
  2. How might you use this information in your work?
  3. How might what you shared with me change with a different type of source, like a website or a journal article or something a doctor said or something in the EHR?

(turn off screen share)

FOLLOW-UP QUESTIONS (10-15 minutes)

1. Please describe moments and or situations in your daily work where you typically need to evaluate information.
   1. What different types of information do you encounter?
   2. What evaluation criteria do you typically apply?
   3. What are the deciding factors for you to either use or ignore the information?
2. Is there anything else you would like to share with me about your evaluation of information?
