# Supplementary material for: “Is this professionally correct?”: understanding the criteria nurses use to evaluate information
Source: J Med Libr Assoc. 2025 Oct 23;113(4):298–309. doi: 10.5195/jmla.2025.2163 (PMC12604069; doi:10.5195/jmla.2025.2163)
Supplement: Supplementary file 4 — Appendix D [file jmla-113-4-298-s04.docx]

**Appendix D: Scenario source characteristics**

**Survey sources for scenario**

**Website**

Swanson, W. S. (2020). Colds and flu: prevention and treatment tips. Knowyourotcs.org.<https://www.knowyourotcs.org/tips-for-cold-and-flu/>

*Excerpt*

**I** always like to start by saying, the flu shot is your best shot at preventing an influenza infection. While it’s best to get immunized early in the flu season, we often continue to recommend getting the flu vaccine well into May. Call you doctor’s office, go to your local pharmacy, or use the CDC’s Vaccine Finder to locate the vaccine available in your zip code. Remember, if you have a baby, they will need two doses (separated by 28 days or more), and if your child is under the age of nine and they have never had the flu vaccine before, they’ll need two doses this year, too!"

**
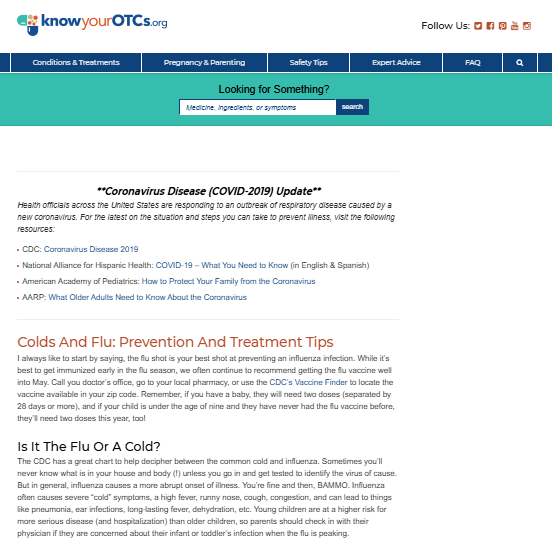
**

*Screenshot from website as it appeared at the time of the survey. Participants only saw this if they clicked the link.*

*Favorable indicators (librarian-determined)*

- Written for lay audience
- Links to authoritative sources
- Aligns with public health guidance
- Common judgment of .org URLs as higher quality

*Unfavorable indicators (librarian-determined)*

- Unclear and possibly obscured sponsorship of information
- Produced by a trade organization that participates in lobbying

**Article**

Cagle, P. J. (2021). Shoulder injury after vaccination: A systematic review. *Revista Brasileira de Ortopedia, 56*(3), 299-306. <https://doi.org/10.1055/s-0040-1719086>

*Excerpt*

"Adverse reactions to vaccine injections are usually mild and incredibly rare in nature, but multiple cases of shoulder events including bursitis, generalized pain or decreased range of motion have been reported following routine vaccine administrations. These events are known as Shoulder Injury Related to Vaccine Administration or SIRVA.

A systematic review of literature was performed to identify all published accounts of SIRVA. Twenty-seven papers reporting one or more accounts of SIRVA were identified. The most common vaccination involved was the Influenza vaccine. The most common symptoms were pain that began in 48 hours or less and loss of shoulder range of motion. The most common treatment modalities were physical therapy, corticosteroid injections and anti-inflammatory medication; but in some patients, surgery was required. Regardless of intervention, the vast majority of outcomes demonstrated improved pain and functional except in the occasions of nerve injury.

The etiology of SIRVA injuries has multiple possibilities including needle length, mechanical injury from needle overpenetration and the possibility of an immune inflammatory response from the vaccine components, but a unique definitive test or quantifiably result does not yet exist."


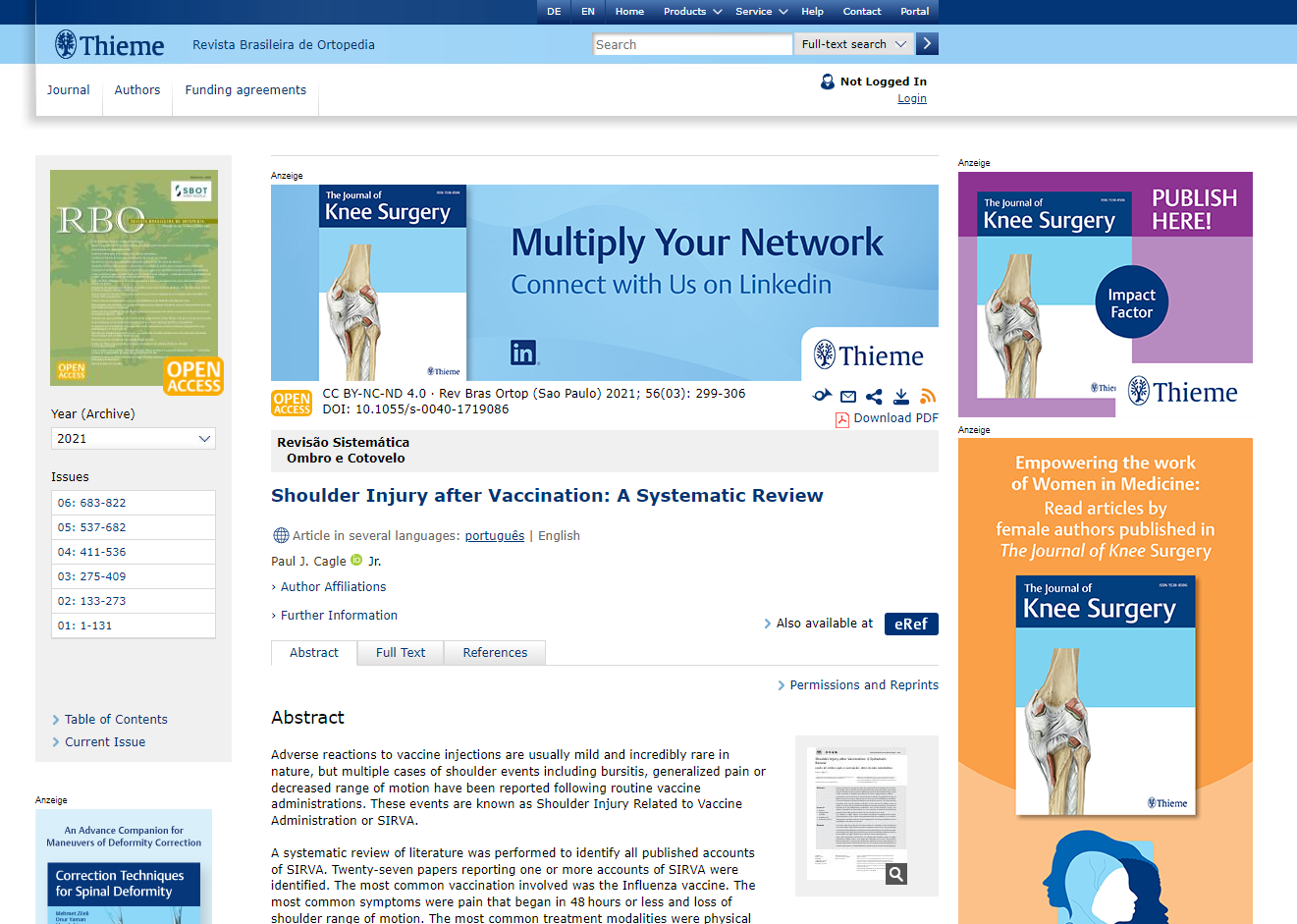


*Screenshot of article as it appeared at the time of the survey. Participants only saw this if they clicked the link.*

*Favorable indicators (librarian-determined)*

- Systematic review
- Peer-reviewed journal
- Author is healthcare professional

*Unfavorable indicators (librarian-determined)*

- Authorship does not meet standards for methodology
- Report of methods does not meet methodological rigor
- Potential bias about international research

**Interview sources for scenario**

**Website**

Well-Being Index Team (2021 Feb 24). The biggest causes of nurse burnout and what you can do. Well Being Index. <https://www.mywellbeingindex.org/blog/the-biggest-causes-of-nurse-burnout-and-what-you-can-do/>

*Excerpt*

"The biggest union of registered nurses in the United States — National Nurses United — describes [nurse burnout](https://web.archive.org/web/20220814092706/https://www.nationalnursesunited.org/news/beating-burnout-nurses-struggle-physical-mental-and-emotional-exhaustion-work) as "physical, mental and emotional exhaustion." Nurse burnout leads to job dissatisfaction and affects patient outcomes. A survey conducted in 2012 revealed that around [one-third of nurses reported](https://web.archive.org/web/20220814092706/http://dpeaflcio.org/wp-content/uploads/nursing-2013.pdf) an emotional exhaustion score (a calculation used to measure psychological fatigue) of 27 or more, recognized by medical professionals as "high burnout." In a separate study, 49 percent of registered nurses under the age of 30 experienced significant levels of exhaustion. But why are so many nurses at breaking point? And how are health care providers dealing with this issue?."

**
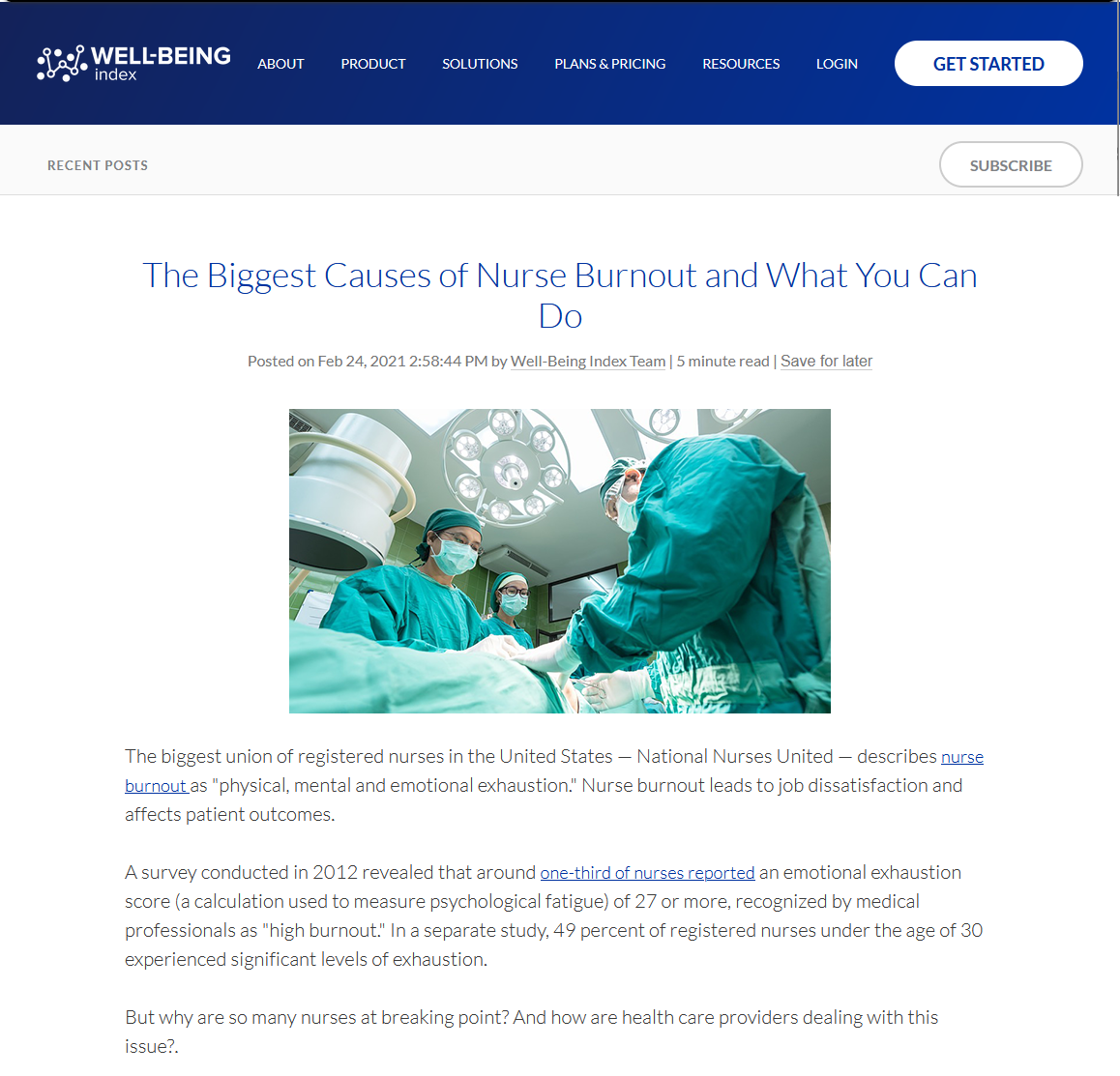
**

*Screenshot of website as it appeared during the interviews, from the Internet Archive Wayback Machine. Participants were shown the whole website and encouraged to ask the librarian to scroll.*

*Favorable indicators (librarian-determined)*

- Written for lay audience
- Links to authoritative sources
- Common judgment of .org URLs as higher quality

*Unfavorable indicators (librarian-determined)*

- Produced by an organization with a likely financial interest

**Article**

Zhou, Q., Lai, X., Wan, Z., Zhang, X., & Tan, L. (2021). Impact of burnout, secondary traumatic stress and compassion satisfaction on hand hygiene of healthcare workers during the COVID-19 pandemic. *Nursing Open, 8*(5), 2551-2557 <https://doi.org/10.1002/nop2.786>

*Excerpt*

Aim: To assess the prevalence of burnout, secondary traumatic stress, and compassion satisfaction and explore their impacts on self-reported hand hygiene among medical aid teams in the COVID-19 period in Wuhan, China. Design: Cross-sectional study. Method: A total of 1,734 healthcare workers from 17 medical aid teams were surveyed. The survey included burnout, secondary traumatic stress and compassion satisfaction measured by the professional quality of life scale and self-reported hand hygiene. Data were collected between 5–7 March 2020. Multiple regression analyses were performed. Results: Burnout and secondary trauma stress were at low and average levels, and compassion satisfaction was at average and high levels. Burnout was negatively associated with hand hygiene, while compassion satisfaction was positively associated. Hospital administrators should pay attention to burnout and compassion satisfaction to improve infection control behaviours. Management of healthcare workers in our study may be constructive in emerging infectious diseases.

**
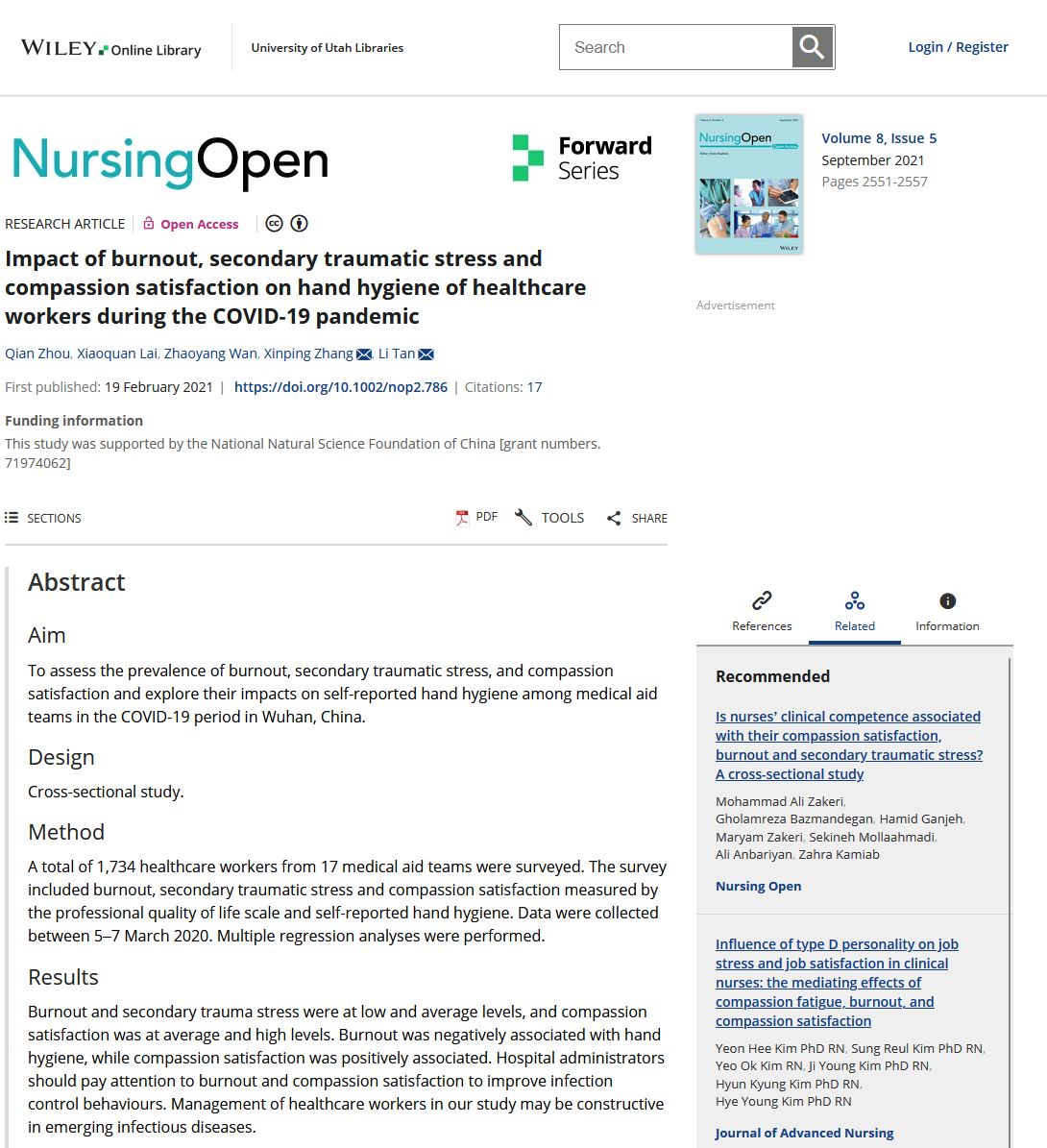
**

*Screenshot of article abstract as it appeared during the interviews. Participants were shown the whole article and encouraged to ask the librarian to scroll.*

*Favorable indicators (librarian-determined)*

- Empirical study
- Peer-reviewed journal

*Unfavorable indicators (librarian-determined)*

- Small sample of dates covered
- Potential bias about international research
