# Supplementary material for: “Is this professionally correct?”: understanding the criteria nurses use to evaluate information
Source: J Med Libr Assoc. 2025 Oct 23;113(4):298–309. doi: 10.5195/jmla.2025.2163 (PMC12604069; doi:10.5195/jmla.2025.2163)
Supplement: Supplementary file 5 — Appendix E [file jmla-113-4-298-s05.docx]

**Appendix E: Codebook**

**Criteria:** A norm, benchmark, or heuristic applied to evaluate information. The information may meet the benchmark or not. Criteria are used to determine suitability of information.

| **Criteria** | **Definition** |
| --- | --- |
| Accuracy | *The correctness of the information (citations present to support claims), how the information came about (experiments, opinions (possible bias), whether the publication went through some sort of editing process and/or peer review. The information matches existing research (see lateral reading under processes) or prior knowledge.* |
| Authority | *The qualifications/education or reputation of author(s) of the information or reputation of journal/publisher/organization that contains or puts out the information.* |
| Currency | *The age of the information. Terminology commonly used by subjects when discussing currency are recency, currency, publication date, or up-to-dateness, etc.* |
| Editorial Quality | *How the study organization, detail, etc. determines whether or not the nurse uses the information.* |
| Methodology | *How the research was done.* Granularity:   - Confounding factors - Levels of evidence - Limitations - Population - Sample size - Statistical analysis - Study design |
| Publisher | *The organization that provides a platform for dissemination of information (includes journal publishers as well as databases like Pubmed)* |
| Purpose | *References to why the information was created (e.g., to sell something, to persuade, to inform, to evoke emotional response) and for which audience.* Granularity:   - Audience - Funding source - Source created to persuade leading to biased information (i.e. ads) |
| Relevance | *The match, or fit, between the research question/information need, and the content of the source. Relevance includes applicability or fit to a specific workplace setting or workplace culture.* |
| Undetermined | *Study participant used criteria but it was not clear what criteria is used.* |
