# Supplementary material for: “Is this professionally correct?”: understanding the criteria nurses use to evaluate information
Source: J Med Libr Assoc. 2025 Oct 23;113(4):298–309. doi: 10.5195/jmla.2025.2163 (PMC12604069; doi:10.5195/jmla.2025.2163)
Supplement: Supplementary file 6 — Appendix F [file jmla-113-4-298-s06.docx]

**Appendix F: Other specialties as indicated by respondents of the survey**

Other specialties:

- Home health
- Post acute
- Primary care house calls; geriatrics
- Pain management
- Endoscopy procedural dept
- Research
- Addiction
- Rehabilitation
- Graduate school MSN
- Neurology
- Occupational health
- Quality
- Adolescent residential treatment
- Nursing education
- Dermatology
- Anesthesia
- Clinical research
- OB/GYN office
- Paramedical examer for life ins. Company
- Insurance in public domain
- Home health diabetes specific
- Outreach education
- Clinical research
- Home care
- Outpatient ENT clinic
- Corporate wellness
- Nurse educator
- Dermatology
- Nurse Education
- Education
- Rehab
- Endoscopy
- Anesthesia
- Hospice
- Remote check-up calls to prevent emergencies
- Family practice
- Neurodevelopmental problems
- Pain management
- Trasport neonatal and pediatrics
- Geriatrics
- Flight nurse
- Endoscopy
- Pain management
- Pain management
- Dialysis
- Orthopedics
- Geriatric nurse
- Home health
- med/surg and surgery
- Surgical services
- Transport
- Clinical Care Float pool
- Same day surgery
- Informatics
- Changing to home health
- STD clinic public health nurse
- Utilication management
- Geriatrics
