# Supplementary material for: “Is this professionally correct?”: understanding the criteria nurses use to evaluate information
Source: J Med Libr Assoc. 2025 Oct 23;113(4):298–309. doi: 10.5195/jmla.2025.2163 (PMC12604069; doi:10.5195/jmla.2025.2163)
Supplement: Supplementary file 7 — Appendix G [file jmla-113-4-298-s07.docx]

## **Appendix G: Statistical Analysis of Survey Data**

It is typical to use the p-value of 0.05 to measure significant differences between values. However, when multiple tests are performed on the same set of data, it increases the risk of a Type I error (false positive). Because multiple statistical tests were performed on this data, we chose to correct the p-value to reduce the risks of a false positive. The simplest way to do this is to use the Bonferroni correction, which divides the typical p-value by the number of tests performed. Because there were 13 possible responses, this results in a cutoff value of .05/13 which is 0.004. A more complicated and less conservative way of adjusting the value is to use the Benjamin-Hochberg critical value. This gives a different p-value for each question based on sorting the p-value for each response and using its ranked value to create the adjusted p-value.

As can be seen in Tables below, though some of the p-values are below the typical significance cutoff of .05, after the p-value is adjusted, none of the results are significant.

**Table 1**

P-Values for Chi-Square Tests on Education Level and Criteria to Evaluate Public Websites

| Question | Pearson Chi Square P-value | Adjusted BH Critical Value Cutoff |
| --- | --- | --- |
| The information is accurate | 0.063 | 0.015 |
| The information is relevant to what I need | 0.803 | 0.042 |
| The information is reported somewhere else | 0.822 | 0.046 |
| The information is in its original context | 0.254 | 0.023 |
| The information's purpose (e.g. presenting facts or opinions) | 0.025 | 0.008 |
| The information is in the most appropriate publication type | 0.906 | 0.050 |
| The information's production and/or dissemination | 0.518 | 0.031 |
| The information fits with what I already know | 0.503 | 0.027 |
| The information is biased toward one point of view | 0.044 | 0.012 |
| The information is current | 0.656 | 0.038 |
| The source's financial backing, financing, or underwriting | 0.117 | 0.019 |
| The authors' expertise | 0.013 | 0.004 |
| None of the above | 0.518 | 0.035 |

**Table 2**

P-Values for Chi-Square Tests on Number of Years Worked as a Nurse and Criteria to Evaluate Public Websites

| Question | Pearson Chi Square P-value | Adjusted BH Critical Value Cutoff |
| --- | --- | --- |
| The information is accurate | 0.882 | 0.046 |
| The information is relevant to what I need | 0.353 | 0.023 |
| The information is reported somewhere else | 0.184 | 0.019 |
| The information is in its original context | 0.041 | 0.008 |
| The information's purpose (e.g. presenting facts or opinions) | 0.404 | 0.027 |
| The information is in the most appropriate publication type | 0.826 | 0.042 |
| The information's production and/or dissemination | 0.991 | 0.050 |
| The information fits with what I already know | 0.558 | 0.035 |
| The information is biased toward one point of view | 0.731 | 0.038 |
| The information is current | 0.041 | 0.012 |
| The source's financial backing, financing, or underwriting | 0.025 | 0.004 |
| The authors' expertise | 0.128 | 0.015 |
| None of the above | 0.426 | 0.031 |

**Table 3**

P-Values for Chi-Square Tests on Education Level and Criteria to Evaluate Scholarly Sources

| Question | Pearson Chi Square P-value | Adjusted BH Critical Value Cutoff |
| --- | --- | --- |
| The information is biased toward one point of view | 0.02 | 0.004 |
| The information is relevant to what I need | 0.048 | 0.008 |
| The source's financial backing, financing, or underwriting | 0.079 | 0.012 |
| The information's production and/or dissemination | 0.146 | 0.015 |
| The information is accurate | 0.183 | 0.019 |
| The information is reported somewhere else | 0.211 | 0.023 |
| The information's purpose (e.g. presenting facts or opinions) | 0.345 | 0.027 |
| The information is in the most appropriate publication type | 0.36 | 0.031 |
| The information is in its original context | 0.37 | 0.035 |
| The information is current | 0.422 | 0.038 |
| The information fits with what I already know | 0.489 | 0.042 |
| The authors' expertise | 0.757 | 0.046 |
| None of the above | 0.92 | 0.050 |

**Table 4**

P-Values for Chi-Square Tests on Number of Years Worked as a Nurse and Criteria to Evaluate Scholarly Websites

| Question | Pearson Chi Square P-value | Adjusted BH Critical Value Cutoff |
| --- | --- | --- |
| The information is accurate | 0.51 | 0.038 |
| The information is relevant to what I need | 0.156 | 0.012 |
| The information is reported somewhere else | 0.6 | 0.046 |
| The information is in its original context | 0.363 | 0.023 |
| The information's purpose (e.g. presenting facts or opinions) | 0.535 | 0.042 |
| The information is in the most appropriate publication type | 0.012 | 0.004 |
| The information's production and/or dissemination | 0.879 | 0.050 |
| The information fits with what I already know | 0.178 | 0.015 |
| The information is biased toward one point of view | 0.398 | 0.027 |
| The information is current | 0.408 | 0.031 |
| The source's financial backing, financing, or underwriting | 0.129 | 0.008 |
| The authors' expertise | 0.184 | 0.019 |
| None of the above | 0.485 | 0.035 |

**Table 5**

Primary Vs. Bedside Nurses and Evaluation Criteria

| Criteria | Public Websites | Scholarly Sources | BH Critical Value |
| --- | --- | --- | --- |
| Accurate | 0.159 | 0.032 | 0.015 |
| Relevant | 0.64 | 0.259 | 0.027 |
| Reported Elsewhere | 0.312 | 0.883 | 0.046 |
| Original Context | 0.849 | 0.036 | 0.019 |
| Information Purpose | 0.633 | 0.504 | 0.038 |
| Appropriate | 0.604 | 0.196 | 0.023 |
| Production/Dissemination | 0.402 | 0.026 | 0.012 |
| Fits Prior Knowledge | 0.142 | 0.997 | 0.050 |
| Biased | 0.044 | 0.023 | 0.004 |
| Current | 0.548 | 0.341 | 0.031 |
| Financial Backing | 0.007 | 0.024 | 0.008 |
| Author Expertise | 0.333 | 0.596 | 0.042 |
| None of the above | 0.095 | 0.449 | 0.035 |

**Table 6**

Administrative Nurses and Evaluation Criteria

| Criteria | Public Websites | Scholarly Sources | BH Critical Value |
| --- | --- | --- | --- |
| Accurate | 0.538 | 0.268 | 0.015 |
| Relevant | 0.097 | 0.102 | 0.027 |
| Reported Elsewhere | 0.364 | 0.012 | 0.046 |
| Original Context | 0.617 | 0.717 | 0.019 |
| Information Purpose | 0.223 | 0.228 | 0.038 |
| Appropriate | 0.029 | 0.096 | 0.023 |
| Production/Dissemination | 0.991 | 0.685 | 0.012 |
| Fits Prior Knowledge | 0.371 | 0.416 | 0.050 |
| Biased | 0.982 | 0.283 | 0.004 |
| Current | 0.375 | 0.079 | 0.031 |
| Financial Backing | 0.402 | 0.122 | 0.008 |
| Author Expertise | 0.109 | 0.694 | 0.042 |
| None of the above | 0.68 | 0.154 | 0.035 |
