# Supplementary material for: “Is this professionally correct?”: understanding the criteria nurses use to evaluate information
Source: J Med Libr Assoc. 2025 Oct 23;113(4):298–309. doi: 10.5195/jmla.2025.2163 (PMC12604069; doi:10.5195/jmla.2025.2163)
Supplement: Supplementary file 8 — Appendix H [file jmla-113-4-298-s08.docx]

**Appendix H: Survey choices & codes consolidated**

| **Survey choice(s)** | **Codebook code(s)** | **Consolidated label** |
| --- | --- | --- |
| Authors' expertise | Authority | Authority |
| Currency | Currency | Currency |
| Production and/or dissemination | Publisher | Publisher, production, or dissemination |
| Relevancy | Relevance | Relevance |
| Accuracy,  Fits Prior Knowledge | Accuracy  Accuracy - Fits Prior Knowledge | Accuracy (including prior knowledge) |
| Purpose,  Bias,  Financial backing | Purpose  Purpose - Funding  Purpose - Persuade | Purpose (includes bias and funding) |
